# Prognostic value of pulmonary vein size in prediction of atrial fibrillation recurrence after pulmonary vein isolation: a cardiovascular magnetic resonance study

**DOI:** 10.1186/s12968-015-0151-z

**Published:** 2015-06-18

**Authors:** Thomas H. Hauser, Vidal Essebag, Ferdinando Baldessin, Seth McClennen, Susan B. Yeon, Warren J. Manning, Mark E. Josephson

**Affiliations:** Cardiovascular Division, Department of Medicine, Beth Israel Deaconess Medical Center and Harvard Medical School, 330 Brookline Avenue, RW-453, Boston, 02215 Massachusetts; Division of Cardiology, McGill University Health Center, Montreal, Canada; Azienda Ospedaliera Treviso, Treviso, Italy; Harbor Medical Associates, South Weymouth, Massachusetts; UpToDate, Waltham, Massachusetts; Department of Radiology, Beth Israel Deaconess Medical Center, Boston, Massachusetts

**Keywords:** Atrial fibrillation, Catheter ablation, Cardiovascular magnetic resonance, Pulmonary vein

## Abstract

**Background:**

The relationship between pulmonary vein (PV) anatomy and successful catheter ablation of atrial fibrillation (AF) is poorly understood

**Methods:**

First-pass contrast enhanced PV magnetic resonance angiography was performed in 71 consecutive patients prior to PV isolation. PV diameter and cross-sectional area (CSA) were measured prior to PV isolation. Any symptomatic or asymptomatic AF >10s was considered a recurrence. Early recurrence was defined as recurrent AF ≤30 days after PV isolation, while late recurrence of AF was defined as recurrent AF >30 days after.

**Results:**

At 1 year, 57 % had any recurrence of AF while 41 % had late recurrence of AF. Study subjects with one or more PV diameter in the top 10^th^ percentile had trend toward more early recurrent AF (HR 1.99, p = 0.053). Study subjects with one or more PV CSA in the top 10^th^ percentile had more late recurrent AF (HR 2.25, p = 0.039) and a trend toward more early recurrent AF (HR 1.94, p = 0.064). With multivariate analysis, PV size was not associated with early recurrent AF, but late recurrent AF was associated with one or more large PV, increased left atrial size, and non-paroxysmal AF. Study subjects with all three of these risk factors had a 100 % rate of late recurrent AF at 1 year, while those with none had a 7 % rate of late recurrent AF.

**Conclusions:**

Larger PV size is independently associated with more late recurrent AF after PV isolation. Determination of PV size prior to PV isolation may predict procedural success.

## Background

Atrial fibrillation (AF) is the most common sustained cardiac arrhythmia [1]. The recognition that the pulmonary veins (PV) have a critical role in the development and maintenance of AF has led to the development of several procedures to electrically isolate the PV from the left atrium to prevent recurrent AF [2–7]. The cause of recurrent AF is incompletely understood but is associated with electrical reconnection of the PV with the left atrium [8, 9]. The extent to which anatomic measurements of the PV might predict electrical reconnection has not been investigated.

We hypothesized that patients with large PV would be at higher risk for electrical reconnection and thus more recurrent AF after PV isolation. We evaluated this hypothesis in a consecutive series of 71 patients who underwent PV magnetic resonance angiography (MRA) prior to PV isolation.

## Methods

### Study cohort

The study cohort was comprised of a consecutive series of 71 patients who underwent cardiovascular magnetic resonance (CMR) prior to PV isolation for the treatment of AF. The clinical characteristics of the study cohort are summarized in Table 1. Antiarrhythmic drug therapy had failed in 58 (72 %) after the use of 1 (29 [41 %]), 2 (19 [27 %]), or 3 or more (10 [14 %]) drugs. The study was approved by the hospital Committee on Clinical Investigations (Institutional Review Board), with waiver of written informed consent.

**Table 1:** Characteristics of the study cohort and hazard ratios for the recurrence of a trial fibrillation (AF)

		Early Recurrent AF		Late Recurrent AF	
Characteristic		HR (95 %)	p	HR (95 % CI)	p
Demographics					
Men	55 (77 %)	3.62 (1.10 – 11.88)	0.034	1.84 (0.64 – 5.32)	0.261
Age, years *	52 ± 11	1.27 (0.92 – 1.75)	0.147	1.01 (0.97 – 1.05)	0.621
Body surface area, m^2^	2.10 ± 0.22	0.68 (0.14-3.39)	0.634	0.36 (0.07 – 1.97)	0.237
Body mass index, kg/m^2^	29.3 ± 5.6	0.99 (0.93 – 1.06)	0.762	0.99 (0.92 – 1.06)	0.780
Type of atrial fibrillation					
Paroxysmal	40 (56 %)	0.36 (0.18 – 0.73)	0.004	0.36 (0.17 – 0.80)	0.012
Persistent	25 (35 %)				
Permanent	6 (8 %)				
Medical history					
Mitral regurgitation †	33 (46 %)	1.68 (0.85 – 3.35)	0.138	1.52 (0.71 – 3.25)	0.280
Hypertension	32 (45 %)	1.27 (0.64 – 2.52)	0.488	1.38 (0.64 – 2.95)	0.409
Obstructive sleep apnea	19 (27 %)	0.79 (0.35 – 1.74)	0.551	1.17 (0.51 – 2.68)	0.713
Diabetes mellitus	10 (14 %)	1.38 (0.57 – 3.36)	0.471	2.04 (0.86 – 4.83)	0.106
Coronary artery disease	7 (10 %)	1.27 (0.44 – 3.61)	0.659	1.54 (0.46 – 5.15)	0.489
Obstructive lung disease	3 (4 %)	0 (not defined) ||	0.991	0 (not defined) ||	0.991
No comorbidity	22 (31 %)	0.71 (0.32 – 1.58)	0.400	0.89 (0.38 – 2.12)	0.799
CMR measurements					
LV ejection fraction, % *	61 ± 10	0.92 (0.66 – 1.28)	0.609	1.22 (0.79 –1.88)	0.371
LV end diastolic volume, ml *	163 ± 40	0.91 (0.82 – 1.01)	0.067	0.92 (0.82 – 1.04)	0.168
LV mass, g *	129 ± 34	1.03 (0.93 – 1.14)	0.611	0.96 (0.86 – 1.08)	0.516
Left atrial dimension (4-chamber), cm	5.7 ± 0.8	1.58 (0.99 – 2.51)	0.056	2.20 (1.38 – 3.49)	<0.001

* The hazard ratio is reported for a 10 unit change

† Determined by the presence of ≥ mild mitral regurgitation on echocardiography

|| No subjects with obstructive lung disease had a recurrence of AF

HR = hazard ratio, CI = confidence interval, LV = left ventricular

### CMR Technique

CMR was performed using a 1.5 T whole-body MR system (Intera, Philips Medical Systems, Best, The Netherlands) with a five-element cardiac synergy coil for radiofrequency signal reception.

First pass breath-hold 3D contrast enhanced MRA of the PV was obtained after manual bolus administration of 0.2 mmol/kg gadopentetate dimeglumine (Magnevist ®, Berlex Laboratories, Wayne, NJ), immediately followed by a saline flush. Data acquisition began after a delay determined by a small timing bolus given prior to contrast enhanced MRA. A spoiled end-expiratory breath-hold 3D gradient echo sequence with the following parameters was used: repetition time 3.6 ms, echo time 1.1 ms, flip angle 30 degrees, 50 slices, slice thickness 4 mm interpolated to 2 mm, field of view 480 mm, matrix 272 × 512, imaging time 22 s. The 3D volume was centered on the left atrium and included all PV. Images were prospectively acquired in the axial plane. Commercially available system software (EasyVision 5.1 or ViewForum R5.1, Philips Medical Systems, Best, The Netherlands) was used to generate multiplanar reformations.

Steady-state free-precession breath-hold gradient echo ECG-gated cine CMR was performed in the 4-chamber and contiguous short axis orientations during a series of end-tidal breath-holds. Short axis images were acquired from the left ventricular base to the apex with 10 mm slices and no gap. The following parameters were used: repetition time 3.0 ms, echo time 1.5 ms, flip angle 60 °, field of view 480 mm, matrix 208 × 256.

### Image Analysis

All measurements were made by an observer blinded to the clinical treatment data using commercially available system software (EasyVision 5.1, Philips Medical Systems, Best, The Netherlands). The maximum left atrial dimension was measured in the 4-chamber orientation. Left ventricular volumes, mass and ejection fraction were determined using Simpson’s rule [10]. The PV were measured at the location in the sagittal plane at which the PV separate from the left atrium and from each other identified by viewing a sagittal plane reconstruction of the dataset [11]. The maximal diameter and cross-sectional area (CSA) of each PV were measured. A left common PV was defined as a single left-sided PV entering the left atrium as determined in the sagittal plane. A right middle PV was defined as any right-sided pulmonary vein identified in the sagittal plane in addition to the right inferior and right superior PV.

Any PV with a diameter in the top 10^th^ percentile of all PV was defined as having a large diameter. Similarly, any PV with a CSA in the top 10^th^ percentile was defined as having a large CSA. A separate analysis was performed for each measure of PV size.

### PV Isolation Procedure

The electrophysiology procedure was performed using a femoral venous approach. A decapolar catheter was positioned in the coronary sinus and a second catheter was placed in the right atrium. Left atrial access was obtained by two transseptal punctures. Transesophageal or intracardiac echocardiography was used to identify PV anatomy and velocities before and after ablation, to identify catheter position with reference to the vein ostia, to determine the presence or absence of pericardial effusion, and in some cases to guide transseptal puncture. Following transseptal puncture, patients received intravenous heparin to maintain a serum activated clotting time >250 s.

Three-dimensional electroanatomic mapping of the left atrium and PV was performed using a non-irrigated 4 or 8 mm tip NaviStar™ catheter (Biosense Webster) and CARTO™ (Biosense Webster) and/or EnSite NavX™ (Endocardial Solutions) recording systems. Electrograms were recorded during sinus rhythm, coronary sinus pacing, or AF at the ostia of the PV with a 10–14 pole circumferential catheter with distal ring configuration (Biosense Webster or Bard). Radiofrequency ablation was performed outside the PV ostium near sites with the earliest PV electrograms. Ablation was performed for 20–60 s with a target temperature of 52 °C. Temperature was considered adequate if it reached 45 °C. The process was repeated until complete bi-directional electrical PV isolation was achieved, defined by both entrance block as demonstrated by loss of PV potentials, and exit block demonstrated by failure to capture the left atrium during sinus rhythm by pacing (at 10 mA and 2 ms pulse width) each of the bipolar pairs of electrodes of the circumferential catheter positioned at the entrance of the PV. All PV were routinely isolated for all patients.

After PV isolation, induction of AF was attempted by burst pacing from the right atrium and coronary sinus before and after administration of isoproterenol. Isolation of the PV was reassessed and if reconnection was observed the vein was re-isolated. Left atrial ablation lines (mitral isthmus line and/or posterior left atrial line) were performed in 16 (23 %) patients with inducible sustained left atrial tachycardia. A right atrial isthmus line (tricuspid valve to inferior vena cava) was performed in 17 (24 %) patients with a history of or inducible right atrial isthmus dependent flutter.

### Post ablation care and monitoring

Patients were treated with warfarin to achieve an international normalized ratio of 2.0 to 3.0 for at least 6 months after PV isolation, as well as acetylsalicylic acid (325 mg/day) for at least 1 month. Antiarrhythmic drugs were continued post procedure for patients with a history of persistent or permanent AF, and were reinitiated in patients with early (<30 days) recurrences of AF. Antiarrhythmic drugs were used in 29 (41 %) patients post procedure, including 12 (29 %) free of AF at last follow-up.

Evaluation of symptomatic or asymptomatic AF was performed using both a 2 week transtelephonic event recorder and 24 h Holter monitor at 1, 3, 6, and 12 months. Additional monitoring was done for patients with symptoms. Recurrent AF was deemed present if an asymptomatic or symptomatic atrial tachyarrhythmia consistent with AF was documented to last >10 s. Early recurrent AF was defined as any recurrent AF during the first 30 days. Late recurrent AF was defined as recurrent AF after 30 days. The median duration of follow-up was 442 days.

### Statistical Methods

Continuous values are reported as the mean ± standard deviation. Categorical values are reported as counts and percentages. The occurrence of recurrent AF was estimated using the product-limit (Kaplan-Meier) method. The relationship between PV size and recurrent AF was assessed using the product-limit method and the log rank test. The relationship of PV size and clinical variables with recurrent AF was assessed using proportional hazards regression. The proportional hazards assumption was confirmed for all variables by examining log-log survival curves. Multivariate analysis was performed using proportional hazards regression with backward selection of variables. Any variable with a p-value of ≤0.1 was eligible for inclusion and retention in the model. Hazard ratios (HR) are reported as the estimate with the 95 % confidence interval (CI). A p-value of <0.05 was used for determination of statistical significance. All statistical analysis was performed using SAS for Windows (v9.3, SAS Institute, Cary, NC).

## Results

The mean duration of the electrophysiological procedure was 257 ± 63 min. The mean number of ablations performed was 73 ± 25 with a mean total ablation time of 53 ± 23 min. There was no relationship between the procedure time, total number of ablations or total ablation time with any measure of PV size or with the presence of one or more large PV by any measurement criteria (p >0.15 for all).

**Fig. 1:**
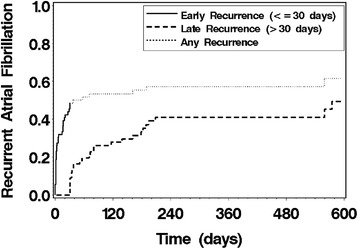
Product-limit estimates for the probability of recurrent atrial fibrillation (AF) for the entire cohort, shown for those with early recurrent AF (recurrence within the first 30 days), late recurrent AF (recurrence after 30 days), or any recurrent AF

At 1 year of follow-up, 57 % had any recurrent AF and 41 % had late recurrence of AF (Fig. 1). The relationship of the clinical characteristics of the cohort with the recurrence of AF are shown in Table 1. There was no relationship of the performance of additional left or right atrial ablation lines with the recurrence of AF (p >0.7 for both). Early recurrent AF was strongly associated with late recurrent AF (HR 5.14 (95 % CI 2.06 – 12.80, p <0.001).

Size measurements for all PV are shown in Table 2. Data regarding the prognostic significance of PV size is shown in Table 3. The relationship of PV size to recurrent AF was examined by evaluating the recurrence in study subjects with one or more large PV as defined by boundary values at the 95^th^, 90^th^, and 85^th^ percentiles of the individual PV measurements. The presence of one or more large PV CSA was associated with both early and late recurrence of AF, while the presence of one or more large PV diameter was associated with early recurrence of AF only. The optimal boundary value for the diameter was the 90^th^ percentile. The 95^th^ and 90^th^ percentile boundary values for the CSA produced similar results for late recurrence of AF. Based on these results, we chose a boundary value of the 90^th^ percentile as the optimal measure to determine that a PV was large. Using this criterion, 24 (34 %) patients had one or more PV with a large diameter and 23 (32 %) had one or more PV with a large CSA. Multiple large PV were present in 2 patients with large diameter PV and 3 patients with large CSA PV. The distribution of large PV is shown in Table 4. Left common and right superior PV were most likely to have increased size while no left superior or right middle PV were large.

**Table 2:** Pulmonary vein measurements

Pulmonary Vein	N	Diameter (cm)	CSA (cm^2^)
Left inferior	58	1.8 ± 0.4	1.7 ± 0.7
Left superior	58	1.7 ± 0.3	1.7 ± 0.5
Left common	13	2.9 ± 0.7	3.4 ± 1.1
Right inferior	71	1.8 ± 0.5	2.3 ± 1.0
Right middle	7	0.9 ± 0.1	0.9 ± 0.2
Right superior	71	2.1 ± 0.6	2.9 ± 1.3
All	278	1.9 ± 0.6	2.2 ± 1.1

CSA = cross-sectional area

**Table 3:** Prognostic value of pulmonary vein size for the prediction of recurrent atrial fibrillation

			Early Recurrent AF		Late Recurrent AF	
Pulmonary Vein Measure	Boundary*	N†	HR (95 %)	p	HR (95 % CI)	p
Diameter						
95^th^ percentile	3.0 cm	8 (11 %)	1.88 (0.77 – 4.56)	0.165	1.28 (0.44 – 3.70)	0.653
90^th^ percentile	2.7 cm	21 (30 %)	1.98 (0.99 – 3.96)	0.053	1.47 (0.67 – 3.21)	0.335
85^th^ percentile	2.4 cm	31 (44 %)	1.87 (0.94 – 3.75)	0.075	1.33 (0.62 – 2.85)	0.464
Cross-sectional area						
95^th^ percentile	4.61 cm^2^	12 (17 %)	2.28 (1.05 – 4.95)	0.037	2.51 (1.05 – 6.00)	0.039
90^th^ percentile	3.71 cm^2^	20 (28 %)	1.94 (0.96 – 3.91)	0.064	2.25 (1.04 – 4.88)	0.039
85^th^ percentile	3.25 cm^2^	30 (42 %)	1.52 (0.77 – 3.01)	0.232	1.98 (0.92 – 4.24)	0.079

*The boundary value marks the percentile boundary for all pulmonary veins individually

† N represents the number of study subjects with at least one pulmonary vein measured ≥ boundary value

**Table 4:** Distribution of large pulmonary veins

Pulmonary Vein	N	Large Diameter (≥2.7 cm)	Large CSA (≥3.71 cm^2^)
Left inferior	58	1 (2 %)	2 (3 %)
Left superior	58	0 (0 %)	0 (0 %)
Left common	13	6 (46 %)	5 (38 %)
Right inferior	71	4 (6 %)	5 (7 %)
Right middle	7	0 (0 %)	0 (0 %)
Right superior	71	17 (24 %)	15 (21 %)

Large pulmonary veins (PV) were defined as PV in the top 10^th^ percentile for each measure of PV size. The percentage of large PV at each position compared to the total number of PV at that position is reported. CSA = cross-sectional area

The relationship between recurrent AF and the presence of one or more large CSA PV was further investigated with multivariate proportional hazards regression (Table 5). With simultaneous adjustment for male gender, the presence of paroxysmal AF, and left ventricular (LV) end diastolic volume, left atrial dimension and one or more large PV CSA were no longer significantly related to early recurrence of AF and were removed from the model. In the analysis of late recurrence of AF, paroxysmal AF, left atrial dimension, and one or more large PV CSA remained in the model after simultaneous adjustment for each the effects of each of these variables. Adjustment for age and gender did not significantly affect these results.

**Table 5:** Multivariate proportional hazards regression for the recurrence of atrial fibrillation

	First Iteration		Second Iteration		Third Iteration	
	HR (95 % CI)	p	HR (95 % CI)	p	HR (95 % CI)	p
Early recurrent AF						
Men	3.31 (0.93 – 11.8)	0.065	3.35 (0.94 – 11.97)	0.063	4.06 (1.17 – 14.04)	0.027
Paroxysmal AF	0.47 (0.22 – 1.00)	0.049	0.44 (0.21 – 0.90)	0.025	0.46 (0.23 – 0.95)	0.036
LV end diastolic volume*	0.87 (0.77 – 0.97)	0.014	0.86 (0.77 – 0.97)	0.011	0.86 (0.77 – 0.97)	0.014
Left atrial dimension	1.14 (0.73 – 1.76)	0.573	Removed		Removed	
One or more large PV CSA	1.72 (0.83 – 3.60)	0.148	1.79 (0.86 – 3.69)	0.118	Removed	
Late recurrent AF						
Paroxysmal AF	0.47 (0.20 – 1.11)	0.086				
Left atrial dimension	1.78 (1.08 – 2.93)	0.023				
One or more large PV CSA	2.07 (0.95 – 4.52)	0.069				

AF = atrial fibrillation, CI = confidence interval, CSA = cross-sectional area, HR = hazard ratio, LV = left ventricle, PV = pulmonary vein.

* The hazard ratio is reported for a 10 unit change

**Fig. 2:**
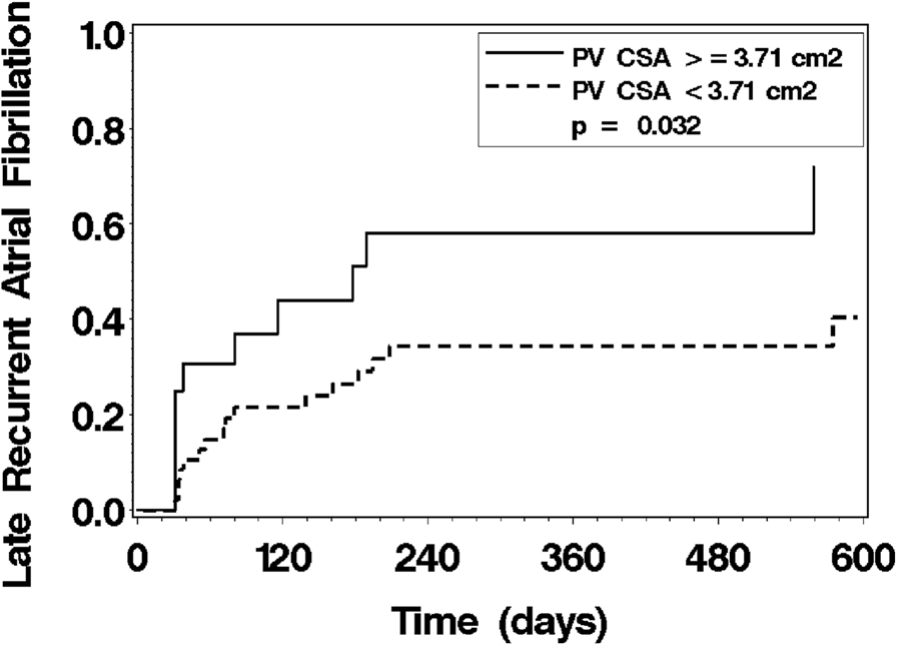
Product-limit estimates for the probability of late recurrent atrial fibrillation stratified by the presence of one or more PV with a cross-sectional area (CSA) ≥3.7 cm^2^. The presence of one or more PV with a cross-sectional area (CSA) ≥3.7 cm^2^ was associated with more late recurrent AF (p = 0.032)

**Fig. 3:**
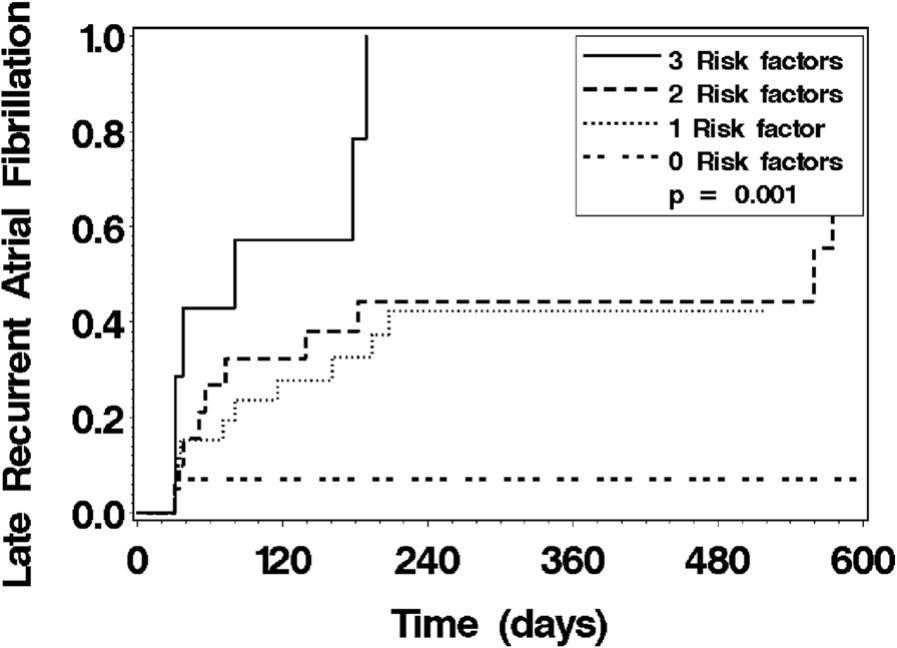
Product-limit estimates for the probability of late recurrent atrial fibrillation (AF) stratified by the number of risk factors present. Risk factors for late recurrent AF were non-paroxysmal AF, left atrial dimension ≥ 5.5 cm, and the presence of one or more PV with a cross-sectional area (CSA) ≥3.7 cm^2^. The presence of more risk factors was associated with more late recurrent AF (p = 0.001)

We further evaluated the late recurrence of AF by stratification of study subjects by the presence of one or more large PV by CSA criteria (Fig. 2), which showed a significant difference between the groups (p = 0.032). Multivariate proportional hazards regression identified 3 significant risk factors for late recurrent AF: non-paroxysmal AF, increasing left atrial dimension, and the presence of one or more large PV CSA. Stratification of the study cohort by the number of these risk factors present identified three risk groups for recurrence (Fig. 3). Those study subjects with all 3 risk factors had a high rate of recurrence (100 % by 1 year). Those study subjects with no risk factors had a very low recurrence rate (7 % at 1 year). Study subjects with 1 or 2 risk factors had intermediate recurrence rates.

## Discussion

In this prospective study of 71 consecutive patients undergoing CMR prior to PV isolation, we found that PV size, left atrial size, and paroxysmal AF are significantly and independently related to late recurrent AF after PV isolation. As PV isolation for the treatment of AF becomes more common, the appropriate selection of patients for these procedures becomes more important. In our study, 100 % of study subjects with non-paroxysmal AF, increased left atrial size, and one or more PV with a CSA in the top 10^th^ percentile had recurrent AF at 1 year while only 7 % of study subjects without these risk factors had late recurrent AF at 1 year.

The PV have a critical role in the pathophysiology of AF. The PV and left atrium are both derived from the primitive common PV [12] and therefore have many anatomic and histologic similarities. Both are smooth-walled structures that have electrically active myocardium. Approximately 90 % of PVs contain atrial myocardium.[13] Although the myocardium in the atrium is uniform, myocardium in the PV is frequently discontinuous and fibrotic. Patients with a history of AF uniformly have myocardium in the PV and an increased rate of structural abnormalities. These structural abnormalities result in abnormal electrical activation with slow and anisotropic conduction that result in proarrhythmic activity [14] that is directly responsible for the generation of AF in many patients [2]. Several catheter-based ablation procedures have been developed to electrically isolate the PV from the left atrium for the prevention of AF with short-term success rates ranging from 65 to 85 % in patients with paroxysmal AF [2–7].

Recurrent AF after these procedures has generally been attributed to recovery of electrical function after the procedure.[8, 9] Larger PV may be at greater risk for electrical reconnection for three reasons. First, larger PV may be more difficult to isolate. Second, a larger perimeter may provide more opportunity for electrical reconnection to take place. Finally, larger PV may have a higher rate of histological and electrophysiological abnormalities that predispose to electrical reconnection. For these reasons, we hypothesized that patients with large PV would be at higher risk for electrical reconnection and thus more recurrent AF after PV isolation. We found that patients with one or more PV in the top 10^th^ percentile for CSA were at increased risk of late recurrent AF.

Paroxysmal AF has been previously described as predicting better outcome after PV isolation compared to persistent or permanent AF. [15–19] We also found that non-paroxysmal AF is independently associated with more recurrent AF. This may be due to left atrial remodeling due to permanent or persistent AF that may produce additional triggers for AF in addition to the PV.

Left atrial size has been previously reported to predict increased rates of recurrent AF, independent of other predictors, [7, 20, 21] a finding that was confirmed in our cohort. A prior study found that left atrial size was a more closely correlated with recurrent AF independent of PV size [21]. We found that both left atrial size and PV size were independently associated with late recurrent AF. This difference may be due to different methods used to measure of left atrial and PV size in the prior report.

Left atrial fibrosis has also been described as a predictor of recurrent AF after PV isolation [22]. We did not routinely obtain pre-ablation late gadolinium enhanced images in this cohort. Prediction of recurrent AF based on the size of the PV appears to compare favorably to prediction based on fibrosis, but assessing both PV size and left atrial fibrosis in patients where both are measured would be required to provide an accurate comparison of predictive accuracy.

We found no other significant relationships of late recurrent AF with any other clinical or anatomic factors. Although advancing age [16], diabetes[23], and the presence of mitral regurgitation [19] have been previously reported as associated with recurrent AF, we found no such relationships. This may be due to the relatively small sample size of this study.

We also examined early recurrence of AF in our dataset and found that male gender, paroxysmal AF, LV cavity size, left atrial size and the presence of one or more large PV were all associated with early recurrence of AF. With multivariate analysis, male gender, paroxysmal AF and LV cavity size remained significantly associated with early recurrence of AF. Male gender [24] and left atrial size [25] have previously been reported to have an association with early recurrent AF. Although prior studies have found a variable relationship of early recurrent AF with late recurrent AF [24–26], we found a significant relationship between the two. The variable findings may be due to different definitions of early compared to late recurrent AF.

We rigorously screened our study cohort for symptomatic or asymptomatic AF using both a 2 week transtelephonic event recorder and 24 h Holter monitor at each follow-up interval as well as additional monitoring for patients with symptoms. All patients with any recurrent AF were deemed a treatment failure even if sinus rhythm was later restored. As a result, our rate of late recurrent AF of 41 % at 1 year is higher than that reported by other investigators [2–7].

## Conclusions

Among patients referred for their initial PV isolation, the presence of one or more large PV CSA, non-paroxysmal AF, and increased left atrial size were independent predictors of late recurrent AF after PV isolation. Stratification by the number of these factors present identified three risk groups. Patients with all three risk factors had a very high rate of recurrent AF. Patients with no risk factors had a very low rate of recurrent AF. The remaining patients had an intermediate rate of recurrent AF. These results suggest that determination of PV size prior to PV isolation may predict procedural success and identify patients for whom the procedure is unlikely to result in long-term success.

## Abbreviations

AF: atrial fibrillation
CI: confidence interval
CMR: cardiovascular magnetic resonance
CSA: cross-sectional area
LV: left ventricle
MRA: magnetic resonance angiography
PV: pulmonary veinv
